# Assessment of the Current Surveillance System for Human Leptospirosis in Ecuador by Decision Analytic Modeling

**DOI:** 10.3389/fpubh.2022.711938

**Published:** 2022-03-03

**Authors:** María Laura Calero, Gustavo Monti

**Affiliations:** ^1^PhD Program in Veterinary Sciences, Faculty of Veterinary Sciences, Universidad Austral de Chile, Valdivia, Chile; ^2^Faculty of Veterinary Sciences, Institute of Preventive Veterinary Medicine, Universidad Austral de Chile, Valdivia, Chile

**Keywords:** leptospirosis, surveillance, Ecuador, surveillance evaluation, public health, epidemiology

## Abstract

Leptospirosis is a globally disseminated zoonotic disease with no national surveillance systems. On the other hand, surveillance is crucial for improving population health, and surveillance systems produce data that motivates action. Unfortunately, like many other countries, Ecuador put in place a monitoring system that has never been tested. The goal of this study was to use scenario tree modeling to assess the sensitivity of Ecuador's current national surveillance system to human leptospirosis as the basis for an economic assessment of the system. We created a decision-tree model to analyze the current system's sensitivity. The inputs were described as probabilities distributions, and the model assessed the program's sensitivity as an output. The model also considers the geographical and weather variations across Ecuador's three continental regions: Andean, Amazonia, and the Coast. Several data sources were used to create the model, including leptospirosis records from Ecuador's Ministry of Public Health, national and international literature, and expert elicitation, all of which were incorporated in a Bayesian framework. We were able to determine the most critical parameters influencing each scenario's output (CSU) sensitivity through sensitivity analysis. The Coast region had the best sensitivity scenario, with a median of 0.85% (IC 95% 0.41–0.99), followed by the Amazonia with a median of 0.54% (CI 95% 0.18–0.99) and the Andes with a median of 0.29% (CI 95% 0.02–0.89). As per the sensitivity study, the most influential criteria on the system's sensitivity were “Attendance or probability of going to a health center” and “probability of having symptoms,” notably for the Coast and Amazonia Regions.

## Introduction

Leptospirosis is a disease caused by infection with at least 64 species of spirochetes from the genus *Leptospira* (1–4). These pathogenic *Leptospira* spp. can infect and cause disease in humans and animals and can be divided into four subgroups (I–IV) (4, 5). It is a worldwide zoonotic disease; nevertheless, it is endemic in Central and South America, where some of the world's highest rates of leptospirosis can be found (6). Despite the fact that it is a prevalent zoonotic disease, it is underreported, and many nations lack surveillance infrastructure (7). The annual burden of leptospirosis is estimated to be 1.03 million cases and 59,800 fatalities (8). Ecuador is an endemic region, and government health records show that the majority of cases are clustered in Manabí province, which is located in the Coast Region, which has favorable weather conditions for the bacteria's growth.

Leptospirosis can cause everything from an undifferentiated fever state to multiple organ failure and death (9). Inadequate diagnosis and monitoring system limitations in most countries have decreased disease awareness within medical and epidemiological communities, contributing to an underestimation of Leptospirosis' burden (10–12). In addition, the low specificity of the symptoms, the diagnostic tests' relative poor diagnostic performance, and the leptospirosis surveillance strategy all had a detrimental impact on case reporting (13). For example, Fontes et al. (14) found that leptospirosis cases in endemic zones of tropical diseases with similar clinical manifestations to leptospirosis were 26–49 times higher than those diagnosed and reported by the Health Services, and other studies have shown the difficulty in diagnosing similar fever syndromes in endemic zones of tropical diseases with similar clinical manifestations to leptospirosis (15, 16).

From a societal perspective, public health surveillance systems should improve the efficiency and efficacy of the public health system, which is a fundamental determinant of population health. By recording data and creating information that public health practitioners and stakeholders may use to improve the quality of their decisions and the effectiveness of their actions, a surveillance system has an impact on population health (17).

Infectious disease surveillance concurrently involves the health care delivery system, public health laboratories, and epidemiologists. The four fundamental components of surveillance are collection, analysis, dissemination, and response, and each of these domains contributes to them. The assessment of a surveillance system should be an approach used by health authorities to evaluate its utility; in other words, the assessment of a surveillance system allows one to estimate the effectiveness and efficiency of the monitoring program, and as a result enhance it. Ecuador, like many other governments, instituted a monitoring system that has never been tested. Epidemiological surveillance, according to Langmuir (18), is the systematic collecting, analysis, interpretation, and timely publication of health data for proper planning, performance, and evaluation of public health programs. The concept of a surveillance system and public health, on the other hand, has evolved over time. Surveillance has a unique meaning in epidemiology: it describes the pattern of disease incidence, it is tied to Public Health, and it studies the natural history of diseases and provides information through baselines (19).

Different aspects of the monitoring system can be examined, and some are more important for specific surveillance goals and health outcomes than others (20). For example, early detection of the disease enables authorities to respond quickly, and as surveillance is one of the most important components of epidemics, early detection of prospective outbreaks and patterns could help prevent the spread of the agent. As a result, evaluating surveillance systems is usually driven by assessing certain qualities. The purpose of mathematical modeling and simulation is to enable for a quick assessment of a surveillance system using data on behavior, transmission routes, and rates of illness and mortality caused by a virus. When collecting data is prohibitively expensive or there are several experimental scenarios to test, simulation is also used (21). Simulation models assist in the prediction and description of complex diseases (22). The Bayesian synthesis approach was proposed by Raftery et al. (23), in which the accessible encoded information about inputs and outputs was encoded in a probability distribution and inferred by constraining this distribution to the submanifold specified by the model. Theoretical foundations of decision analytic modeling can be found in statistical decision theory and anticipated utility theory, and it has functioned as a framework for clinical decision-making, for example Drummond et al. (24). The probability and expected values are essential components of this analysis. Due to its ability to analyze costs and implications, decision analytical modeling (DAM) is gaining traction in this industry. DAM is a framework for combining evidence from various sources and extending the time horizon beyond the follow-up phase (24).

Scenario trees were employed in probabilistic modeling to estimate the sensitivity of a complex non-random component of a surveillance system. It is defined as the likelihood of finding at least one infected person in a population with a specified design prevalence (25, 26). The goal of this study was to determine the sensitivity of Ecuador's current national surveillance system to human leptospirosis using scenario tree modeling as the foundation for an economic evaluation of the system. The sensitivity of the program is critical in generating a comprehensive health economic evaluation of the leptospirosis surveillance system.

## Materials and Methods

### Description of the System

“Health is a right guaranteed by the State, the realization of which is linked to the exercise of other rights, including the right to water, food, education, physical culture, work, social security, healthy environments, and others that sustain food living,” the Ecuadorian Constitution states in Article 32. Furthermore, Article 36 states, “The State, through the national health authority, will be responsible for framing national public health policies, that is, the government provides health, so the national surveillance system covers any individual seeking proper care through Community Health Units (CHU) or district/regional/national referral hospitals.” As a result, Ecuador has a well-developed national health system. All residents, regardless of income or lack of medical insurance, have access to free medical treatment (provided by a comprehensive system of hospitals and regional health clinics). This system also serves remote rural areas, with physicians, dentists, and nurses required to complete a 1-year “rural” residency servicing isolated or underserved populations.

In Ecuador, leptospirosis is a notifiable disease, and the government has set up a passive surveillance system that is coordinated by the Ministry of Public Health across the country. There are two types of Leptospirosis case definitions. The first is a “suspect case,” who is defined as someone who has a fever and at least two other symptoms such as headache, chills, myalgia, stomach discomfort, skin hemorrhage, or altered consciousness with or without jaundice. Furthermore, any risk factor should be exposed to the patient prior to the onset of symptoms, such as exposure to water sources that may be contaminated with rodent and other animal urine, as well as occupational risk activities.

The second is a “confirmed case,” who is essentially a suspected case with a positive microagglutination test (MAT) result. A commercial indirect ELISA test (Panbio™ *Leptospira* IgM ELISA) can be used to quickly screen a suspicious case (for qualitative detection of IgM antibodies to *Leptospira* in serum), and it can be repeated 7 days apart based on the practitioner's criteria. However, as a confirmatory test, an ELISA positive result should be validated by a single MAT during the second or third week. The reciprocal titer for being considered a positive result is 1:100. The testing panel included the 25 serovars recommended by the WHO (for the description of serovars see Supplementary Table 1). Physicians attending to the patient report all cases in this system to the Ministry of Health.

Once the case has been identified, the patient freely receives from health authorities the prescribed treatment like an antibiotic therapy (Doxycycline) a weekly dose of 200 mg for as long as needed, and amoxicillin or erythromycin in pregnant women and children under 8 years of age. The health officers will start an epidemiological investigation to identify possible outbreaks and to trace other contacts. These definitions were established by the Ecuadorian Ministry of Public Health through the official protocol to notifiable disease, which were considered to build the model below.

### Model Description

There are various stages to create a decision-analytic model: (a) Define the problem; (b) Define the model's bounds; (c) Conceptualize the model; (d) Implement the decision tree; and (e) Evaluate the model critically.

**Figure 1:**
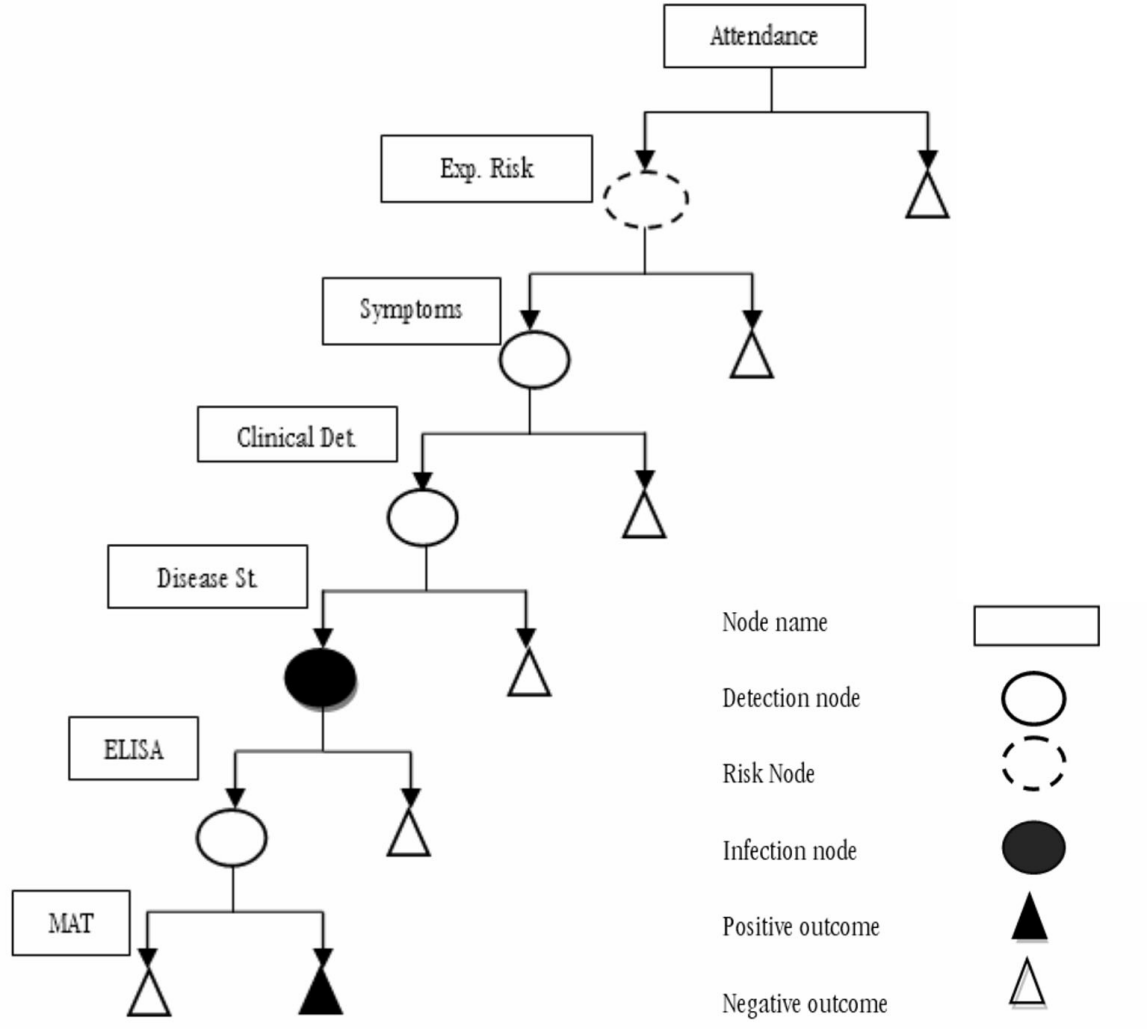
Scenario tree model representing the current national human leptospirosis surveillance system in Ecuador.

We developed a decision-tree model that considered the Epidemiological Surveillance Guidelines (SIVE-Alerta) from the Ecuadorian Ministry of Health's official protocol for notifiable diseases to analyze the current leptospirosis surveillance system's sensitivity in Ecuadorian humans (Figure 1). Also, we identified several parameters based on this protocol (Table 1). The model's inputs were expressed as probability distributions, and the model's output was an estimate of the current program's sensitivity. The model also considers the geographical and weather variations between Ecuador's three continental regions: Andean (Scenario 1), Amazonia (Scenario 2), and Coast (Scenario 3). Table 2 shows the differences in two parameters: “Clinical Detection” and “Prevalence” (status disease).

**Table 1:** List of the current leptospirosis surveillance system component in Ecuador's humans, from nodes and branches to be estimated.

**Nodes**	**Branches**
Attendance to health center	No
	Yes
Expositional risk	No
	Yes
Fever and symptoms	No
	Yes
Clinical detection	No
	Yes
Disease status (prevalence)	Non-infected
	Infected
ELISA	Negative
	Positive
MAT	Negative
	Positive

**Table 2:** Probabilities from specific surveillance system components (disease status and clinical detection) for different geographical areas, of the current surveillance system for human leptospirosis in Ecuador.

**Surveillance system component**	**Scenario 1** **(Andean)**		**Scenario 2** **(Amazonia)**		**Scenario 3** **(Coast)**	
	**Values**	**Dist**.	**Values**	**Dist**.	**Values**	**Dist**.
Disease status (prevalence)	(α= 1.31; β= 5.89)	Beta	(α= 9.27; β= 44.44)	Beta	(α= 27.11; β= 75.31)	Beta
Clinical detection	0.25	Fixed	0.50	Fixed	0.75	Fixed

### Inputs Estimation

#### Data Collection

The decision-tree model was built using a variety of sources (Figure 1), including leptospirosis records from Ecuador's Ministry of Public Health database, national and international literature, and expert elicitation. We used the Delphi method elicitation process to get information from experts. We generated probability distributions for the specific inputs produced by this method, and for other parameters, we reviewed data from the literature and summarized it using a meta-analysis, as indicated below.

##### Delphi Survey

Expert knowledge elicitation (EKE) is the process of extracting information from one or more experts. Experts might be asked for specific information (facts, statistics, sources, requirements, and so on) or their opinions on various topics (preferences, utilities, probabilities, estimates, etc.). In this study, we were primarily interested in expert estimation of uncertain quantities (as facts were found in the literature); in this case, we do not simply want the “best guess” of the quantity, but also a representation of the uncertainty surrounding any guess.

Delphi is a widely used and accepted approach for gathering expert knowledge in specific fields. The fundamental goal of this protocol is to combine the opinions of experts who evaluate a specific topic; in this process, experts make individual estimates and get anonymous feedback until they reach a predetermined level of agreement (27). Finally, the expert opinion is gathered through anonymous questionnaires filled out by the panel (28).

We divided the process in three stages: (1) Preelicitation, (2) elicitation, and (3) postelicitation.

##### Stage 1: Preelicitation

###### Recruit Experts.

We made use of two criteria for identifying experts. First, we looked for professionals who had prior expertise with Leptospirosis clinical and diagnostic detection, sanitary management, or epidemiological research. Second, we balanced the number of experts according to sectors, including government services, academia, and practitioners.

The panel of experts was chosen in multiple steps: initially, we identified a list of experts who were categorized into specialties such as researchers, academics, physicians, health authorities, and others. The residency countries were diverse, although Ecuador was home to most of the experts. The experts were then invited to participate in the survey by e-mail, and the Delphi survey procedure was implemented on the internet.

A total of 15 experts were invited to participate in the study and six declined the invitation. As a result, we assembled a panel of nine experts to interview to analyze our planned leptospirosis surveillance program representing government services (22.2%), practitioners (33.3%), and academia/laboratories (55.5%).

###### Develop Elicitation Questions.

The Delphi protocol obtains quantitative judgments and establishes a confidence interval around the best estimate (29). Each session's questions are targeted toward eliciting probabilities. Experts submit their upper and lower bounds for the lowest and highest chance that an event will occur in this way before eliciting their best estimate. Finally, experts provide their best assessment of the frequency of occurrence of an event.

Experts were requested to determine the plausible minimum value (step 1), feasible maximum value (step 2), and best estimate (step 3) for the surveillance system that made up the various parameters of the model that represented it in each scenario.

##### Stage 2: Elicitation

###### Round of Individual Estimates.

We scheduled an introduction meeting with each expert, in which we explained the expert elicitation process' motives, expectations, and context.

Experts were instructed *via* a videoconference that they could download from YouTube, and as part of the elicitation process, they were issued an electronic questionnaire by e-mail. In the first round, we gathered information such as the probability of each system parameter's minimum, maximum, and most likely values.

##### Stage 3: Postelicitation

###### Aggregate Experts' Judgments.

From expert's individual responses, we obtained probabilities distributions for the parameters related to the surveillance system using a mathematical aggregation approach, giving an equal weightage to all the experts.

Through the Delphi survey, we obtained the information about each parameter or node of the Leptospirosis Surveillance System (Table 1) except for “prevalence level” and “clinical detection parameter” that were categorized by region, and this is explained at the coverage of the model.

##### Metaanalysis

This metaanalysis aimed to derive realistic estimates for these nodes or parameters: “fever and symptoms” and “expositional risk” using existing data on leptospirosis surveillance systems. In addition, as detailed below, the metaanalysis results were combined with the Delphi survey data.

The preferred reporting items for systematic reviews and metaanalysis (PRISMA) criteria were used to conduct the metaanalysis (30). In the English language, platforms such as “Google Scholar,” “Sciences Direct,” “Web of Sciences,” and “Medline” were used as literature sources and searching machines to recover published articles. The publications that were examined were those that were published between 2001 and 2018. To search the literature, we used terms like “leptospirosis,” “spirochetes,” “surveillance,” “assessment,” and “sensitivity,” as well as a Boolean query that combined terms like “leptospirosis surveillance,” “Assessment of leptospirosis surveillance,” “Sensitivity of leptospirosis system,” and “Assessment of spirochetes surveillance.”

**Figure 2:**
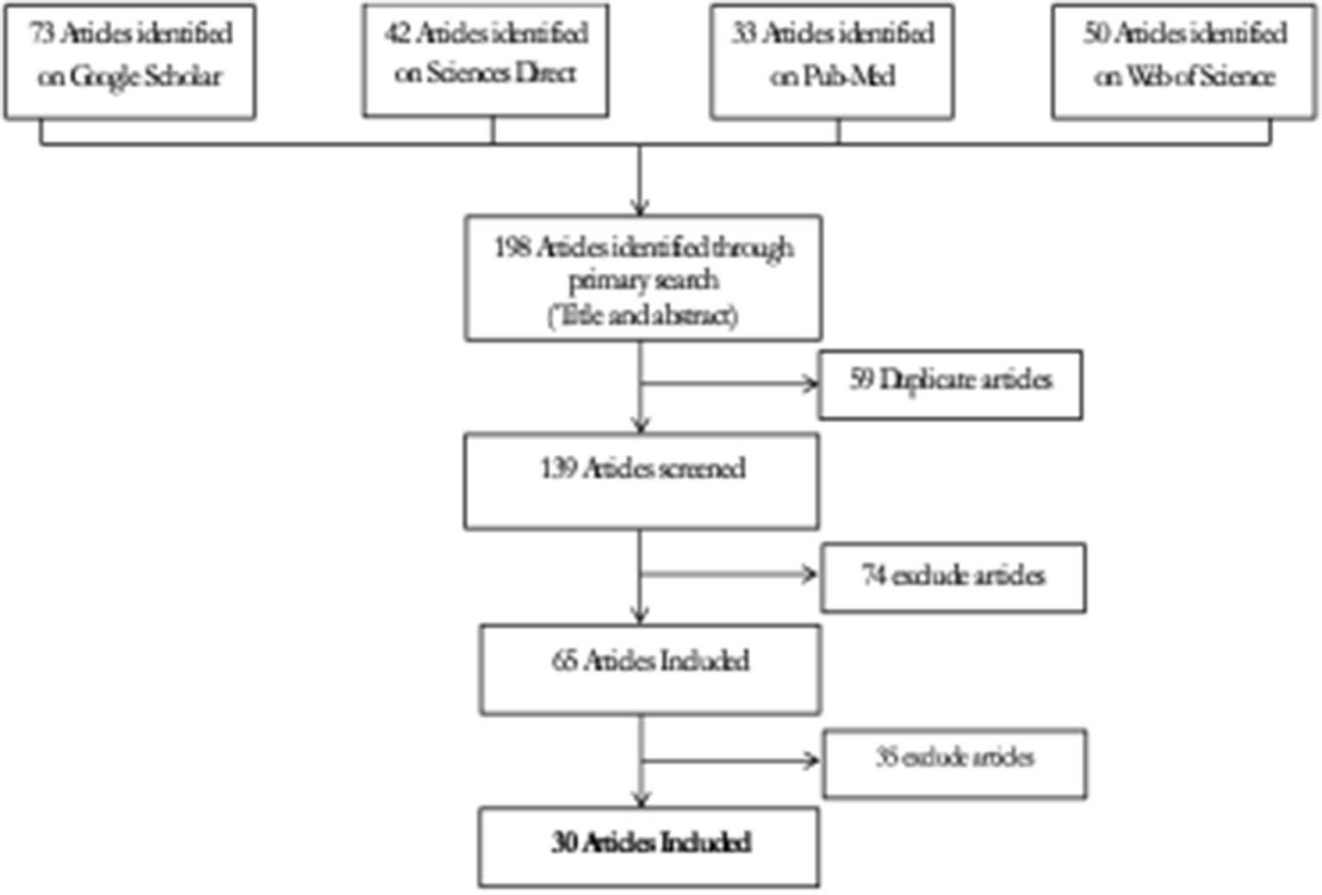
Flow chart diagram of studies selection process in the metaanalysis.

The phases of the systematic review that followed PRISMA criteria were identification, screening, eligibility, and inclusion, as indicated in Figure 2.

For research selection and data abstraction, the selection criteria were first applied to the titles and abstracts of publications before moving on to the whole text. Articles that did not include at least one of the following terms: public health, animal health/disease; theoretical studies without empirical data; and articles with insufficient information to allow evaluation of the method described and without evaluation of the surveillance system described were all excluded. In addition, studies were considered for this study provided they included data from estimating at least one surveillance system attribute.

Data abstraction results were displayed using forest-plots, and publication bias was addressed through funnel plots. We used a metaregression technique to conduct a metaanalysis of the relevant literature. This method enables us to look into patterns of heterogeneity in our data and determine what causes them. The variable x in meta-regression stands for study characteristics. A metaregression model uses this data to try to predict y, the study's effect size. The fact that effect sizes are utilized as predictor variables, however, adds a layer of complexity. By using a mixed-effects model, metaregression achieves this. This model accounts for the fact that observed studies deviate from the true overall effect due to sampling error and between-study heterogeneity. Furthermore, one or more variables x are used to forecast variations in genuine effect sizes.

Then the combined results using the Shelf package (31) from R Software (32) were used to determine the value of each parameter.

For the parameters “fever and two additional symptoms” and “expositional risk,” we evaluated them using two datasets derived from the manuscripts retrieved and selected during the searching process: one of them included all the patients included in the manuscripts (dataset A) and the other just the number of positive *Leptospira* patients (dataset B).

##### Statistical Analysis of Data

The probability distribution captures what is known about the variable, making expert elicitation with priors a logical match for Bayesian techniques (33). As a result, in this study, we emphasize the use of Bayesian models to include expert opinion. In brief, Bayesian modeling consists of four key elements: a prior probability distribution that captures prior knowledge about a parameter; data on the parameters captured by the likelihood; a model that describes the underlying process and incorporates both the likelihood and priors; and posterior estimates that result from combining the likelihood with the prior and reflect model uncertainties (34, 35).

Probabilistic modeling aims to capture the uncertainty in the decision model's input parameters and explain what this means for the model's outputs (36). In a Bayesian model, for example, expert subjective judgments are employed to calculate uncertainty. This uncertainty could be caused by a lack of data, natural variance, or a combination of both (37).

The tree's nodes' values were calculated independently of one another. Then, using the method proposed by Christensen et al. (38) and implemented in the package EpiR (39), run on R Software (V. 3.5.1), we elicited a beta distribution from each expert for each parameter of the existing system (32). The expert information was then combined into conjugate priors, which were then used to represent it.

The posterior distribution was obtained by combining the findings of the metaanalysis with official Department of Health statistics (according to the parameter). It was estimated using the R program MCMCpack (40).

##### Scenario Tree Model Description

A tree represents all the events influencing the detection of the infection as nodes dividing the population into groups of individuals with similar probabilities of being infected and detected. Each of these category nodes may have one or more possible outcomes, with a specific probability of occurrence estimated from historical data, published findings, or expert opinion.

A scenario tree displays the disease detection process using a surveillance system component (SSC), tracing the chances that a single unit (person) will yield a positive or a negative result. To accomplish this, we constructed a stochastic scenario tree model. Scenarios are realizations of multidimensional stochastic processes (trajectories). A scenario tree's root node is connected to multiple endnotes by an SSC, which is a logical succession of states and occurrences. The edge weights are usually determined by probabilities. One of the advantages of this instrument is its ability to analyze infection risks and detection probabilities. Stochastic scenario tree models were built for the quantitative evaluation of complex surveillance systems, and they were used to describe each SSC and evaluate the sensitivity of each SSC, as discussed below. The structure of the scenario tree is described in Figure 1, and the content is described in Tables 1, 2.

Given that the population is sick, the scenario tree separates the population into smaller groups, each with a equal likelihood to become ill. It describes the population's structure as well as any events (inside an SSC) that influence the likelihood of a disease or disease agent being detected by the SSC if one exists. At each branch of the tree, probabilities for each possible outcome are first estimated. Then, by multiplying the probabilities along each branch of the tree and adding those that result in a positive result, SSC sensitivity is calculated (disease detected). Finally, as indicated in the preceding section, the parameters were estimated using metaanalysis and expert elicitation.

From 10,000 runs, we estimated the median and percentiles 5th and 95th for each tree using a Monte Carlo technique. We used the Monte Carlo method to generate the scenario trees in Excel, fixed them with a seed number, and then ran the models stochastically using @RISK (Decision Tool, version 6). We defined input and output parameters as distributions to account for the uncertainty of the estimations.

##### Coverage's and Scope's Model

The sensitivity of an SSC to a population is influenced by the disease's prevalence; the method allows different probability of detection (at the threshold prevalence) to be assigned to different population segments. Thus, the scenario trees coupled geographic risk (three scenarios based on Ecuador's continental areas) with the clinician's ability to notice a suspicious case, which are both expressed in the model's “inputs” parameters, “Status Disease or Prevalence,” and “Clinical Detection,” respectively. Based on reports from official health statistics, we established the geographic risk scenarios for these characteristics (Table 2).

For each scenario, we identified these geographical regions as zones with low (1–10%), medium (11–20%), and high (21–30%) prevalence levels, based on the information from cases reported by health authorities as well as the environmental conditions in each region, which play a key role in *Leptospira* transmission dynamics. We estimated the parameters shape 1 and shape 2 from beta distribution for each scenario based on these assumptions; on the other hand, we assumed that the “clinical detection” parameter could be measured subjectively, so we decided to express it as a fixed value, but we assumed that the level of the zone's prevalence was linked to the doctor's knowledge of the disease (Table 2).

The model's scope was disease detection, with individuals as the unit of analysis, each region's population as the coverage, and a 1-year time frame to account for the length of the intervention and its long-term effects.

##### Component Sensitivity Calculations

The scenario tree modeling method is being used to evaluate the sensitivity of detection, defined as the likelihood of detecting at least one positive case of leptospirosis for a given prevalence by SSC or each type of test defined. Different probabilities of detection (at the threshold prevalence) can likewise be assigned to different population sections using the scenario tree.

The tree is based on the probability of detecting an infected patient (CSeU, SSC unit sensitivity) and is determined by assessing the possibility that any randomly selected unit in the population will provide a positive result (SeU). The component's sensitivity (CSe) is the sensitivity of the surveillance (CSe). It was calculated by adding the branch probabilities for all branches with positive outcomes and computing the total branch probability for each branch of the tree (i.e., for each outcome/terminal node). Finally, using the formula SSe, the total sensitivity of the surveillance system was determined.

#### Evaluation of Influential Parameters per SSC

Through sensitivity analysis, we determined the most important parameters determining the sensitivity of the result (CSeU) for each scenario. @RISK was used to evaluate it (Decision Tool, version 6). Furthermore, this data assists decision-makers in identifying potential avenues for increasing the entire monitoring system's quality.

## Results

### Experts' Elicitation

The outcomes of the expert's elicitation procedure utilizing the Delphi technique are summarized in Table 3. Except for the test sensitivities, the parameter estimations were broad and heterogeneous in general. However, for all parameters, confidence intervals are positive, and therefore, statistically significant.

**Table 3:** Mean probability and their 95% confidence interval of the surveillance system component (SSC) inputs of the current surveillance system for human leptospirosis in Ecuador, based on expert's elicitation using a Delphi method.

**SSC**	**Mean (95% CI)**	**Distribution**
Attendance to health center	0.40 (0.06; 0.80)	Pert
Expositional risk	0.68 (0.21; 0.88)	Pert
Fever and symptoms	0.55 (0.15; 0.96)	Pert
Clinical detection	Fixed value*	
Prevalence	According Scenarios*	
ELISA	0.86 (0.77; 0.95)	Pert
MAT	0.83 (0.68; 0.96)	Pert

* *From Table 2*.

### Metaanalysis Results

**Figure 3:**
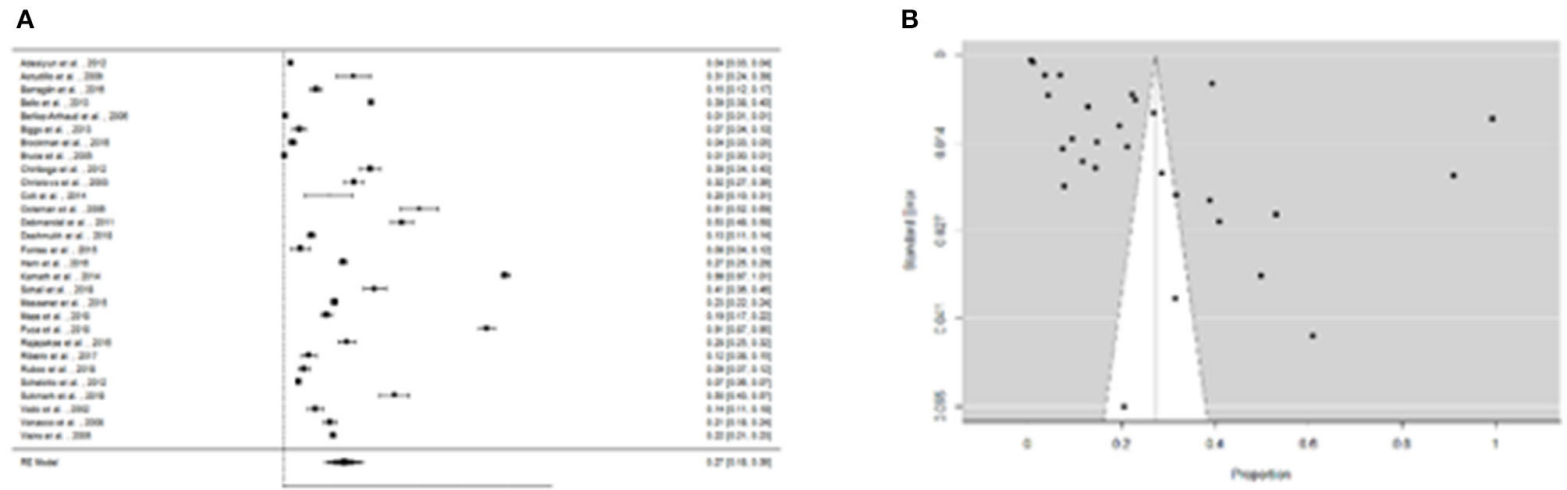
**(A)** Forest plot of the proportion of “fever and two more symptoms” for suspicious patients of leptospirosis. **(B)** Funnel plot of the proportion of “fever and two more symptoms” for suspicious patients of leptospirosis.

**Figure 4:**
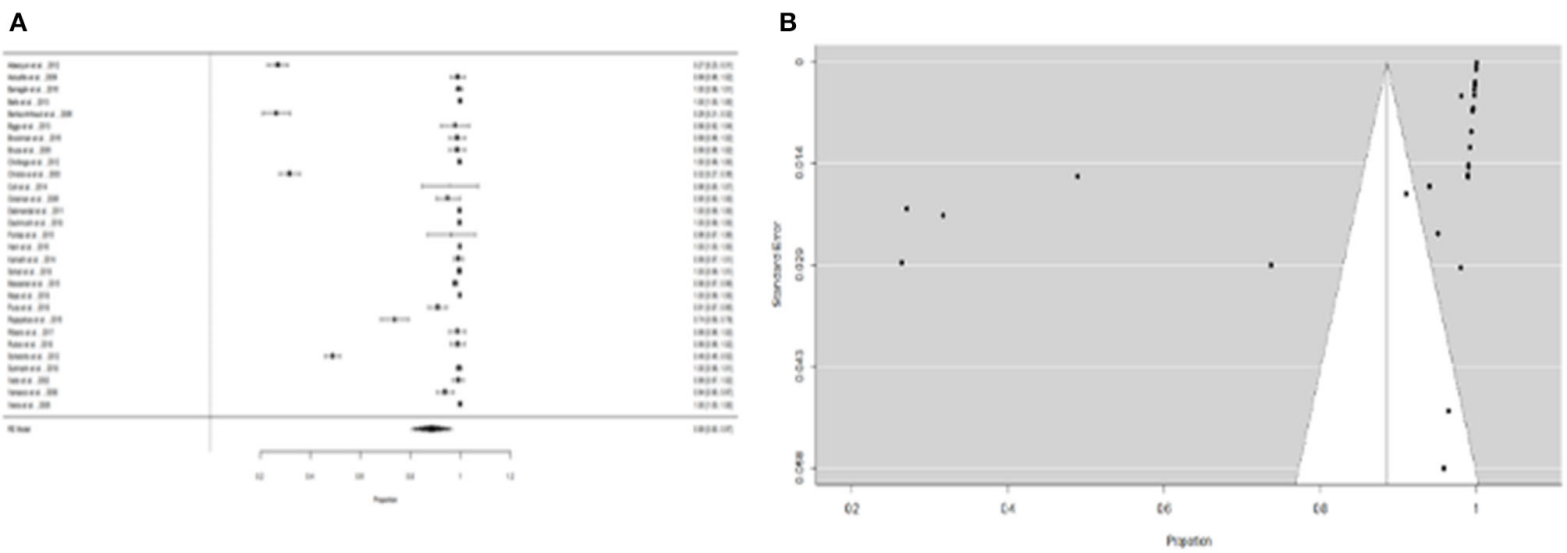
**(A)** Forest plot of the proportion of “fever and two more symptoms” for leptospirosis patients. **(B)** Funnel plot of the proportion of ”fever and two more symptoms" for leptospirosis patients.

We were able to extract 30 studies for the metaanalysis from the 198 publications found (Figure 2). The likelihood of fever plus two additional symptoms for suspicious patients was calculated to be 27% (95% CI = 18–36%) using the whole dataset A (Figure 3). Figure 3B suggests a low probability of publication bias. Furthermore, in confirmed *Leptospira* patients, the likelihood of “fever plus two more symptoms” was calculated to be 89% (95% CI = 80–97%) (see Figure 4A). Figure 4B suggest the presence of potential publication bias. The metaregression estimation and heterogeneity analysis are summarized in Table 4. The results of this table (confidence intervals and model indicators like tau2 or I2) suggest that none of the parameters considered in the analysis explained the estimated heterogeneity.

**Table 4:** Summary of metaregression analysis outputs for the variables that influenced the proportion of fever and two more symptoms at suspicious patients of leptospirosis.

**Variable**	**Estimate**	**95% Confidence** **interval**	***P*** **-value**
Intercept	1.2645	(0.6342; 1.8949)	<0.0001
Cross-sectional design	−0.2147	(−0.6397; 0.2103)	0.3220
Longitudinal design	−0.2850	(−0.6905; 0.1205)	0.1683
Asia	0.0753	(−0.2258; 0.3764)	0.6241
Europe	−0.0876	(−0.4038; 0.2286)	0.5871
Latin-American and Caribbean	−0.0106	(−0.2890; 0.2678)	0.9404
Serologic test	−0.1091	(−0.5745; 0.2656)	0.5719
Serologic and molecular test	−0.1544	(−0.5745; 0.2656)	0.4711
*N*	−0.0001	(−0.0001; 0.0000)	0.0642
Positive *Leptospira* patients	0.0002	(−0.0000; 0.0003)	0.0743

*tau^∧^2 (estimated amount of residual heterogeneity): 0.0544 (SE = 0.0178)*.

*tau (square root of estimated tau^∧^2 value): 0.2333*.

*I^∧^2 (residual heterogeneity/unaccounted variability): 99.92%*.

*H^∧^2 (unaccounted variability/sampling variability): 1201.95*.

*R^∧^2 (amount of heterogeneity accounted for): 0.00%*.

**Figure 5:**
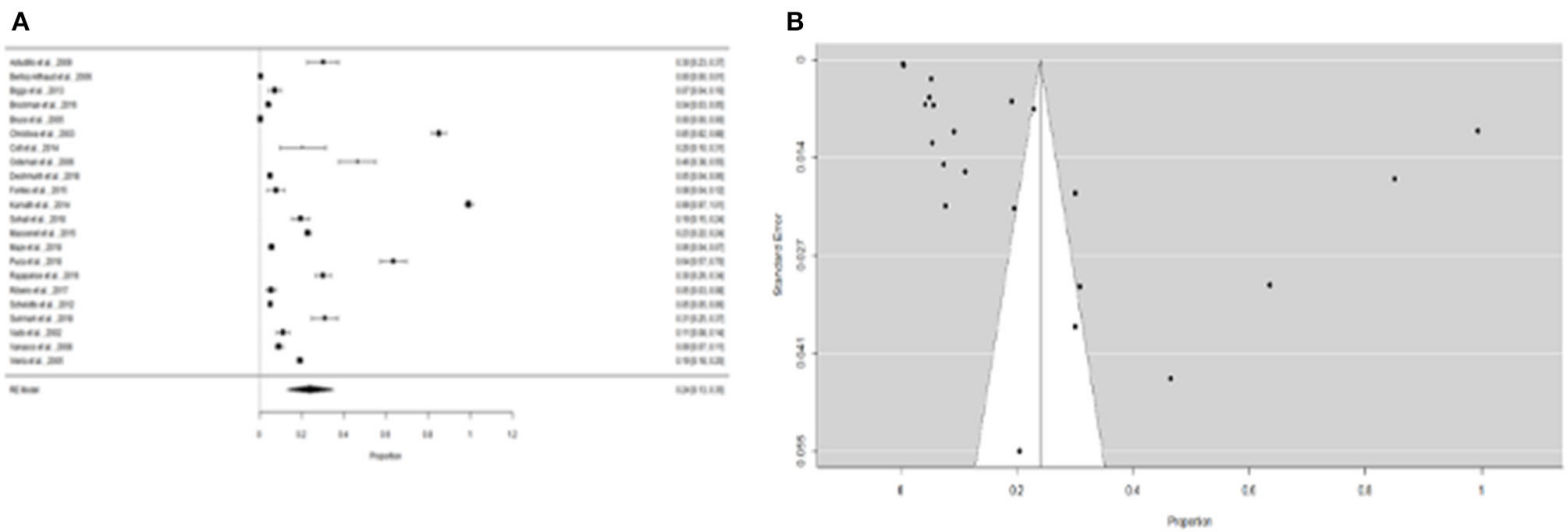
**(A)** Forest plot of the proportion of “expositional risk” for suspicious patients of leptospirosis. **(B)** Funnel plot of the proportion of “expositional risk” for suspicious patients of leptospirosis.

**Figure 6:**
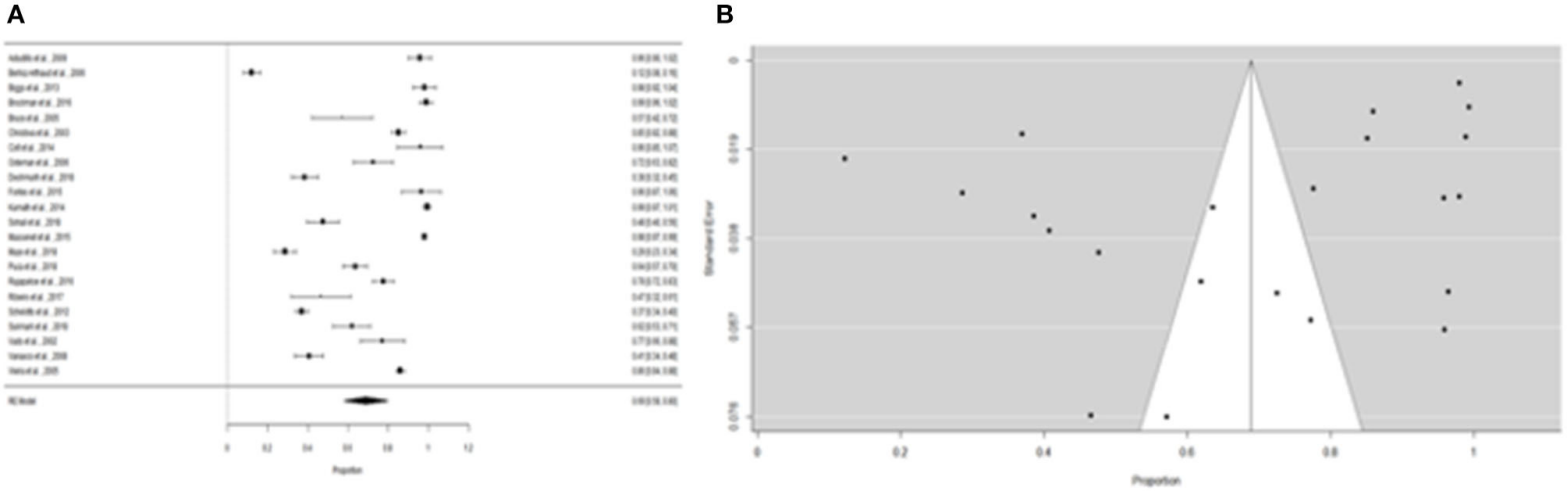
**(A)** Forest plot of the proportion of “expositional risk” for leptospirosis patients. **(B)** Funnel plot of the proportion of “expositional risk” for leptospirosis patients.

Furthermore, the proportion of people with “expositional risk” records was estimated to be 24 percent (95% CI =13–35%) using the more restricted dataset B (Figure 5A). Figure 5B suggests a low probability of publication bias. The estimated proportion of people with “expositional risk” was 69% (95% CI = 58–80%) among confirmed *Leptospira* cases (Figure 6A). Figure 6B also suggests a low probability of publication bias. Table 5 summarizes the metaregression estimation and heterogeneity analysis. The table's results (confidence intervals and model indicators such as tau^2^ or I^2^) indicate that none of the parameters evaluated in the analysis explained the estimated heterogeneity.

**Table 5:** Summary of metaregression analysis outputs for the variables that influenced the proportion of fever and two more symptoms at suspicious patients of leptospirosis.

**Variable**	**Estimate**	**95% Confidence** **interval**	***P*** **-value**
Intercept	1.2112	0.6162; 1.8063	<0.0001
Cross-sectional design	−0.4977	−0.9996; 0.0042	0.0519
Longitudinal design	−0.4417	−0.9374; 0.0540	0.0807
Asia	−0.1736	−0.5625; 0.2153	0.3817
Europe	0.1286	−0.2859; 0.5431	0.5431
Latin-American and Caribbean	−0.0075	−0.3493; 0.3342	0.9655
Serologic and molecular test	0.1421	−0.1940; 0.4783	0.4073
*N*	−0.0001	−0.0002; −0.0000	0.0117
Positive *Leptospira* patients	0.0002	−0.0004; 0.0009	0.4356

*tau^∧^2 (estimated amount of residual heterogeneity): 0.0649 (SE = 0.0260)*.

*tau (square root of estimated tau^∧^2 value): 0.2547*.

*I^∧^2 (residual heterogeneity/unaccounted variability): 98.69%*.

*H^∧^2(unaccounted variability/sampling variability): 76.14*.

*R^∧^2(amount of heterogeneity accounted for): 9.69%*.

*Test for Residual Heterogeneity: QE(df = 13) = 1239.4682, p-val < 0.0001*.

*Test of Moderators (coefficient(s) 2:9): QM(df = 8) = 10.1535, p-val = 0.2544*.

### SSC Inputs Estimation

Table 3 summarizes the resulting input (Mean and CI 95%) probabilities obtained for each SSC that conforms to the current leptospirosis surveillance system in the humans in Ecuador. These values were estimated through the prior probabilities obtained by the Delphi Survey using expert's elicitation.

### Scenario Tree Model

**Figure 7:**
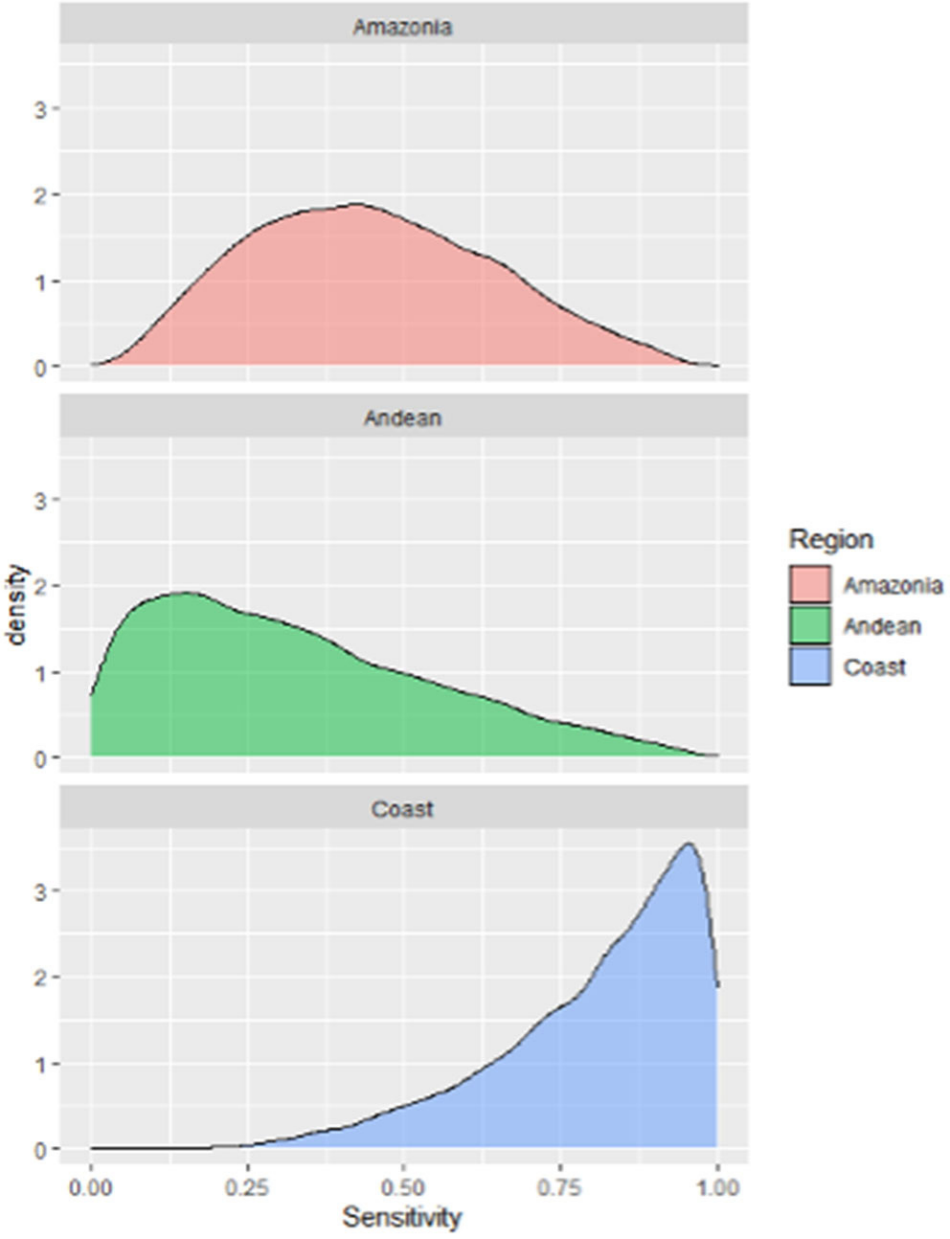
Probability density distributions for the sensitivity of the current surveillance system for humans leptospirosis by different Ecuador geographical scenarios.

The median, 5th and 95th percentiles of each SSC's output distributions for the unit component sensitivity (CSeU-depending on the probability of infection) are presented in Table 6 and Figure 7 and specified by region. The best sensitivity scenario corresponded to the Coast region with a median of 0.85% IC 95% (0.41–0.99) followed by the Amazonia with a median of 0.54% (CI 95% 0.18–0.90) and lastly, the Andes with a median of 0.29% (CI 95% 0.02–0.89).

**Table 6:** Percentiles for surveillance system unit sensitivity and surveillance system component sensitivity, for different geographical scenarios, of the current surveillance system for human leptospirosis in Ecuador.

**Components sensitivity**	**Scenario 1** **(Andean region)**			**Scenario 2** **(Amazonia region)**			**Scenario 3** **(Coast region)**		
	**Percentiles**			**Percentiles**			**Percentiles**		
	**5th**	**50th**	**95th**	**5th**	**50th**	**95th**	**5th**	**50th**	**95th**
CseU	0.0002	0.003	0.02	0.002	0.008	0.02	0.005	0.02	0.05
Cse	0.02	0.29	0.82	0.18	0.54	0.90	0.41	0.85	0.99

*CseU, Surveillance System Unit Sensitivity; CSe, surveillance system component sensitivity*.

### Evaluation of Influential Parameters per SSC

**Figure 8:**
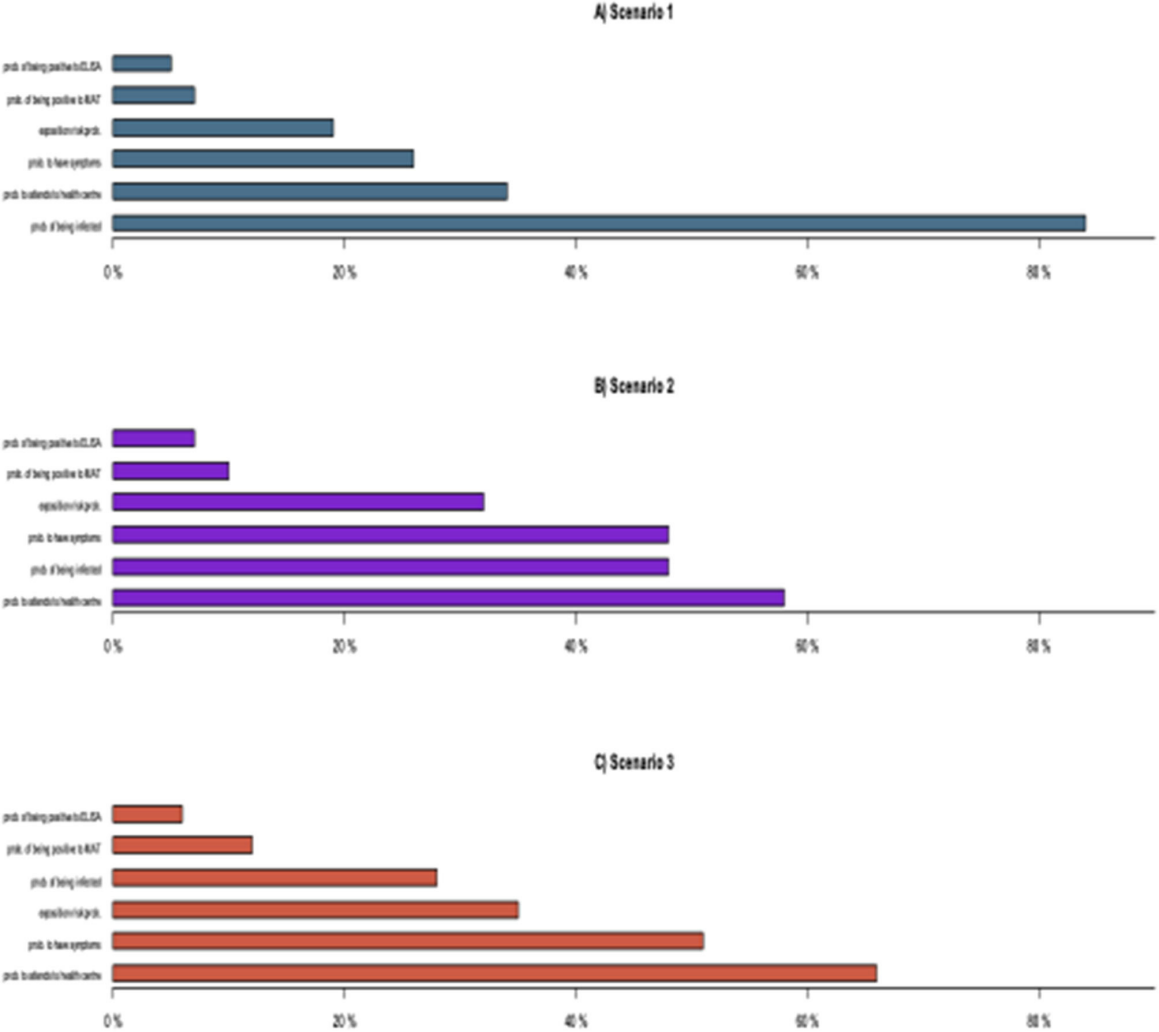
Probabilistic sensitivity analysis. **(A)** For scenario 1 (Andean Region); **(B)** for scenario 2 (Amazonian Region); **(C)** for Scenario 3 (Coast Region).

Figures 8A–C shows the probabilistic sensitivity analysis (PSA), which shows the impact of each SSC on the overall sensitivity of the scenario. Figure 8A depicts the leptospirosis surveillance system's sensitivity analysis for Scenario 1 (Andean Region). The most influential SSC in the model is the “probability of being infected” (Prevalence) (0.84). “Attendance or probability of attending a health center” and “probability to have symptoms” (0.34 and 0.26, respectively) were two other SSCs with a moderate influence, followed by “probability of exposition risk” (0.19). Finally, “probability of being positive to MAT test” and “probability of being positive to ELISA” both do not represent a significant influence on the overall sensitivity of the model (0.07) and (0.05).

Figure 8B shows that the Attendance or “probability to attending a health center” is the most influential SSC in scenario 2 (Amazonia Region), followed by the other two SSC “prevalence or probability to being infected” and “probability to have symptoms” which influences equally (0.48), and “exposition risk probability” (0.32). Again, neither the “probability of being positive to MAT test” nor the “probability of being positive to ELISA” (0.10 and 0.07, respectively) has a significant impact on the model's overall sensitivity.

Finally, Figure 8C shows that “attendance or probability of attending a health center” (0.66) is the most influential SSC in the model for Scenario 3 (Coast Region), and “probability of having symptoms” (0.51), “probability of exposition risk” (0.35), and “prevalence or probability of being infected” (0.28) are also important SSC for this scenario. In other scenarios, the diagnostic test does not have any effect on the overall sensitivity of the system.

## Discussion

The sensitivity of the current national human leptospirosis monitoring system was assessed using a stochastic technique, which is an important attribute of any surveillance system (25). Furthermore, decision-tree modeling offers a transparent and systematic approach to decision-making, as well as chances to improve the framework by obtaining more accurate data (26). With this method, it is possible to include multiple sources of information in the same tree. It is a considerable advantage when not all the data required are available, and expert opinion must be sought. An evaluation of the surveillance activity efficiency provides for a comparison of the performance of various surveillance strategies. To aid in the quantitative assessment of surveillance sensitivity and disease likelihood, the data can be acquired from a range of sources, including random or non-random surveillance data, as well as the documentation of risk differences (41). This is the first evaluation of a human leptospirosis surveillance system that we are aware of, and therefore there is not much to compare it to. However, scenario tree modeling has been frequently used to assess and improve surveillance systems for different illnesses, with most of these assessments concentrating on animal productivity and health (26, 42–44).

Based on an examination of current surveillance-system assessment choices, the model's conclusions will help Ecuador public health policymakers to provide guidance on the basis of national surveillance system for Leptospirosis. Furthermore, by identifying the most relevant features of the selected SSC in relation to the provided CSe, particular attempts to reinforce these SSCs may be implemented.

Human leptospirosis surveillance in Ecuador is a passive system, therefore it is shaped by several circumstances. A case must have at least two or more parameters or symptoms to be reported. The passive component of the monitoring system operates in the same manner as the country. Ecuador, on the other hand, is split into four distinct geographical regions. As a result, disease patterns vary depending on the location. The coast and amazon regions have favorable environmental conditions for pathogenic *Leptospira* spp. transmission, which agrees with our findings that the sensitivity of Ecuador's current national surveillance program for human Leptospirosis is not uniform across regions; this is consistent with the health report, which states that these zones account for most cases. One disadvantage of a passive system is that it ignores these risk differences; as a result, we approached this study's evaluation with the differences between nation regions in mind. We noticed differences in the system's sensitivity between them. The Coast area had the best sensitivity scenario, with a median of 85%. As a result, these communities may be more equipped to recognize outbreaks and the typical course of a leptospirosis case. Furthermore, it implies that the majority of patients are discovered and can be treated if necessary. The sensitivity of the system for the other scenarios is much lower (the Amazonia region has a median sensitivity of 54%, while the Andean region has a median of 29%), and the probability ranges are much wider than for the Coast region. Although it is difficult to determine whether these sensitivities are higher or lower than those of similar monitoring programs in other countries, reaching 100% sensitivity in practice is nearly impossible (45). These findings may arouse concern among authorities because they suggest that there is likely to be a significant under-detection, implying that the disease's burden could be much greater. However, just because the surveillance system's sensitivity is lower in various geographical regions, it does not mean it i's useless; it can still be utilized to track trends as long as the sensitivity is constant (46). Other variables not investigated in this study also compensate for the system's limited sensitivity and keep it operational.

For surveillance systems to have the greatest impact, it is critical to identify system stakeholders, define their roles, and engage them throughout the process (47). The most significant aspects of the system's sensitivity, according to the sensitivity analysis done on the models, were “Attendance or probability of attending a health center” and “probability of having symptoms,” especially for the Coast and Amazonia Regions. As a result, the sensitivity of a system with a higher prevalence and clinical detection will be good, but it will be dependent on the patients' attendance at the health center and, as a result, on whether the patients feel sick and exhibit clinical symptoms that are severe or harmful enough to persuade them to see a doctor. On the one hand, community education should develop trust and confidence among the population by involving typical stakeholders, such as public health practitioners, health care providers, policymakers, and people of affected communities. Medical staff training, on the other hand, can be improved continuously.

Another important metric was “prevalence,” or the likelihood of becoming infected, but only for the Andean Region. These findings are in line with animal disease studies (48), which discovered that the sensitivity of a monitoring system is dependent on the severity of disease in the population. The infection level is determined by the threshold prevalence, which differs from common prevalence; the scenario tree allows for variable chances of being identified (at the threshold prevalence) (25). These data confirm that the Andes' weather conditions are less favorable for the bacterium's survival, and that it has the fewest known cases. The findings could also explain why in hotspot areas of the country, the scenario with reduced sensitivity and existing system restrictions is desirable. Standardization of the leptospirosis threshold prevalence will be critical in the future, and the differences could make sensitivity comparisons of surveillance programs difficult (49).

Non-specific symptomatology characterizes leptospirosis; studies reveal that clinical diagnosis and diagnostic procedures, particularly in developing countries, are still in need of improvement, and physicians frequently confuse leptospirosis symptoms with those of other diseases (50–52). Another important factor we identified was the relationship between the severity of illness in a certain zone and the health professional's knowledge of the condition. Other research shows that physicians should be aware of the characteristics of leptospirosis to avoid confusion when dealing with suspected cases, and that the infection phases should not be overlooked (53). As a result, clinicians should be urged to increase their leptospirosis clinical suspicion (52).

We included the parameter “clinical detection or illness detection capacity” in the model since we considered it as an important aspect of the surveillance system and wanted to explore how critical it was. Due to a lack of information on the value for that variable, the subjectivity of this parameter, and the variability in physician's abilities to diagnose the disease correctly among patients across the country, we built different subscenarios combining different conditions as low, medium, and high clinical detection capacity applied on a population of patients with a low, medium, and high prevalence of the disease. For example, one scenario stated that clinicians in a low-prevalence zone had limited ability to recognize questionable patients, and so on. Medical students lack comprehension of zoonoses, according to research (54), and there is a link between relative knowledge of different diseases and the frequency or recognition in everyday practice (55).

Furthermore, the occurrence (or absence of) of an illness in a selected area is likely to alter awareness (56), and the ability to detect suspicious cases in endemic areas could be a problem. As a result, as has been shown during arbovirus outbreaks (57, 58), it may increase leptospirosis mortality, given that symptoms are similar but misinterpretation occurs and many cases are either not treated or diagnosed too late to establish a prompt therapy. As a result, the next step in defining a zoonotic surveillance system's sensitivity should be to parameterize the “capacity of clinical detection” or healthcare practitioners' awareness of zoonotic infections.

One of the study's drawbacks is the variability, uncertainty, and bias in the sources of information on inputs. Although the methodology we used allowed us to account for variability and uncertainty in the data we handle through input distributions, there was a lack of precise and unbiased information on different parameters; as a result, we are likely to have obtained inaccurate estimates, which could affect the surveillance program's sensitivity estimate (59). The model was constructed by combining data from a literature review and parameterizing it using expert estimates; however, it is not without risk.

Scenario tree models do not account for the time gap between nodes or parameters, which is a weakness in this approach (26, 60). The temporal frame used in an economic evaluation is the duration of health outcomes and costs. The nature of the disease and intervention under consideration, as well as the analysis' purpose, all influence the time span used for economic modeling (York; 2016). The incubation period of the disease and the consequences of the intervention strategy determine the model's time frame; as a result, we publish the findings in this context, following the routine of epidemiological reports conducted by the health authority. Other studies looked at the incubation period that was dependent on the characteristics of the disease (48, 49, 61). According to Martin et al. (25), the amount of surveillance data acquired over time, and thus the SSC's estimated sensitivity over time, has no effect on the projected chance of the population being free at threshold prevalence. Time is not explicit in this model and has no bearing on sensitivity, in contrast to the population scope in the estimate; in this study, we decided to input data based on several scenarios.

The surveillance system's sensitivity is the most important factor for early detection, and scenario tree modeling is a crucial tool for developing effective surveillance strategies (62). According to the World Health Organization, governments should review surveillance programs while pursuing early detection of epidemics, optimize them in a period of public resource scarcity, and underestimate cases (WHO). All of these factors capture our attention and underline the need to assess Ecuador's national human leptospirosis surveillance system and, if possible, propose a new strategy to address the system's inadequacies. Health authorities in the Americas, on the other hand, must improve notification. The leptospirosis record, for example, is an important tool for highlighting the disease's importance as a public health issue and should be registered as an official document. Leptospirosis is underreported in many countries and territories, and incomplete records prevent the disease from being recognized as a public health issue. As a result, it is necessary to start documenting leptospirosis baselines across the Americas, enhance laboratory capacity, and, eventually, harmonize case definitions across countries (7).

This study is the first of its kind in the world on the effectiveness of a human leptospirosis surveillance system, and the methodology might be applied to other disease surveillance systems. Systematic surveillance system evaluation is also essential to aid decision-making processes or the implementation of various surveillance strategies. Furthermore, concluding the system's evaluation with a cost-effectiveness analysis is important for making more holistic selections, allowing for efficient monitoring systems that might save authorities a large amount of money (63). Government resource allocation is a prevalent problem (especially in developing countries), and more work should be done to carry out cost analyses to improve the effectiveness and sustainability of monitoring programs.

## Conclusions

Based on scenario tree modeling, we give the first assessment of the sensitivity of Ecuador's current national Leptospirosis surveillance system for humans. As a result of this research, several qualitative insights have been gathered. The present Ecuador human Leptospirosis monitoring system, for example, differs in sensitivity across the country's many areas, and we were able to quantify the relative contributions of each SSC in the system.

The current surveillance system is sensitive enough to be utilized for decision support and performance monitoring in high-risk locations (coastal area); however, it may present far more cases than the system reports in other parts of the country. As a result, surveillance system evaluation should be conducted as a complementary strategy by public health practitioners and other surveillance system stakeholders. Despite the difficulties, monitoring approaches can still be improved.

## Data Availability Statement

The raw data supporting the conclusions of this article will be made available by the authors, without undue reservation.

## Ethics Statement

The studies involving human participants were reviewed and approved by the Ministry of Health, Ecuador. The experts participants provided their written informed consent to participate in this study.

## Author Contributions

MC and GM contributed to the conception and design of the study, substantially contributed to the discussion of the results, provided critical feedback, and contributed to the writing of the manuscript. MC collection of the data and the statistical analysis. All authors contributed to the article and approved the submitted version.

## Funding

This research was supported by PHD fellowship (#21170784) of Agencia Nacional de Investigación y Desarrollo (ANID), Chile.

## Conflict of Interest

The authors declare that the research was conducted in the absence of any commercial or financial relationships that could be construed as a potential conflict of interest.

## Publisher's Note

All claims expressed in this article are solely those of the authors and do not necessarily represent those of their affiliated organizations, or those of the publisher, the editors and the reviewers. Any product that may be evaluated in this article, or claim that may be made by its manufacturer, is not guaranteed or endorsed by the publisher.
