# Supplementary material for: Assessment of the Current Surveillance System for Human Leptospirosis in Ecuador by Decision Analytic Modeling
Source: Front Public Health. 2022 Mar 3;10:711938. doi: 10.3389/fpubh.2022.711938 (PMC8927665; doi:10.3389/fpubh.2022.711938)
Supplement: Supplementary file 1 [file Table_1.DOCX]

# Supplemental material

**STable 1.** *Leptospira* species, reference strains, serovars and serogroups included in the Micro Agglutination Test result (MAT) for leptospirosis case confirmation.

| **Species** | **serovar** |  | **Species** | **serovar** |
| --- | --- | --- | --- | --- |
| *L. interrogans* | Icterohaemorrhagiae |  | *L. noguchii* | Panama |
|  | Canicola |  | *L. kirschneri* | Grippotyphosa |
|  | Pomona |  |  | Cynopteri |
|  | Australis |  | *L. weilii* | Celledoni |
|  | Autumnalis |  | *L. biflexa* | Patoc |
|  | Pyrogenes |  | L. *santarosai* | Shermani |
|  | Hebdomadis |  | *L. borgpetersenii* | Hardjo |
|  | Bataviae |  |  | Javanica |
|  | Wolfii |  |  | Sejroe |
|  | Copenhageni |  |  | Tarassovi |
|  | Castellonis |  |  | Saxkoebing |
|  | Djasiman |  |  |  |
|  | Bratislava |  |  |  |
